# Variation in rates of early development in *Haliotis asinina *generate competent larvae of different ages

**DOI:** 10.1186/1742-9994-9-2

**Published:** 2012-02-17

**Authors:** Daniel J Jackson, Sandie M Degnan, Bernard M Degnan

**Affiliations:** 1 School of Biological Sciences, University of Queensland, St. Lucia 4072, Queensland, Australia; 2 Courant Research Centre Geobiology, Georg-August University of Göttingen, Goldschmidtstr.3, 37077 Göttingen, Germany

**Keywords:** Developmental variation, Developmental timing, Invertebrate, Competence, Heterochrony, Metamorphosis, Dispersal, Plankton, Larva

## Abstract

**Introduction:**

Inter-specific comparisons of metazoan developmental mechanisms have provided a wealth of data concerning the evolution of body form and the generation of morphological novelty. Conversely, studies of intra-specific variation in developmental programs are far fewer. Variation in the rate of development may be an advantage to the many marine invertebrates that posses a biphasic life cycle, where fitness commonly requires the recruitment of planktonically dispersing larvae to patchily distributed benthic environments.

**Results:**

We have characterised differences in the rate of development between individuals originating from a synchronised fertilisation event in the tropical abalone *Haliotis asinina*, a broadcast spawning lecithotrophic vetigastropod. We observed significant differences in the time taken to complete early developmental events (time taken to complete third cleavage and to hatch from the vitelline envelope), mid-larval events (variation in larval shell development) and late larval events (the acquisition of competence to respond to a metamorphosis inducing cue). We also provide estimates of the variation in maternally provided energy reserves that suggest maternal provisioning is unlikely to explain the majority of the variation in developmental rate we report here.

**Conclusions:**

Significant differences in the rates of development exist both within and between cohorts of synchronously fertilised *H. asinina *gametes. These differences can be detected shortly after fertilisation and generate larvae of increasingly divergent development states. We discuss the significance of our results within an ecological context, the adaptive significance of mechanisms that might maintain this variation, and potential sources of this variation.

## Introduction

The study and comparison of metazoan developmental programs has provided biologists with many insights into the ways in which evolution acts to generate morphological novelty. For example, minor differences in the genetic instructions that regulate development can yield an adult phenotype that differs significantly from a closely related species (for examples see [1-3]). In our studies with the tropical abalone *Haliotis asinina *(a marine gastropod with a biphasic life cycle and planktonically dispersing larvae), we frequently observe considerable variation in rates of development within a cohort of synchronously fertilised gametes [4], a phenomenon that can be observed in many species of indirect developing invertebrates [5].

For many benthic marine invertebrates, dispersal is achieved by a planktonic larval phase that may or may not feed [6-10]. Factors that can affect the length of time spent in the plankton and thus dispersal potential [11,12] may include variation in larval size [13], growth [14], maternal investment [11,15] and behaviour [16,17]. Variation in dispersal and larval recruitment are in turn well known to be mechanisms that can structure adult populations [18,19]. Related to these observations is the fact that many marine invertebrate larvae must be exposed to specific and 'patchily' distributed chemical or physical cues in order for the processes of settlement and metamorphosis to be initiated [20-22]. These cues must often be encountered during a certain developmental state known as 'competence' in order for the cue to engender a response [23-25]. Variation in the rate of embryonic and larval development during the dispersal phase, and therefore in the time to acquire competence, therefore has the potential to affect the likelihood of encountering a suitable metamorphosis inducing cue in a patchy environment. Interestingly, it has long been known for some species that the rate of development is under genetic control. In *Drosophila melanogaster *developmental rate has a selectable and heritable component [26-30]; for example after 125 generations of selection for faster development, Chippendale et al. established two lines of fruit flies that completed larval development 25-30% faster than their non-selected controls/ancestors [27]. This acceleration in developmental time came at a cost, with pre-adult survivorship reduced by more than 10% relative to controls [27]. More recent studies have identified some of the genes (for example *Merlin *and *Karl*) linked to this phenotypic variation [31]. However it is clear that for *Drosophila *developmental timing is a complex trait influenced by many pleiotropic genes and environmental interactions. In the first attempt to do so with a marine invertebrate, Hadfield [32] reported that after 27 generations of intense selection, lines of faster and slower developing nudibranchs (*Phestilla sibogae*) could not be generated. Due to the paucity of data, cross species comparisons of the molecular mechanisms that underlie developmental timing are not yet possible, however it is clear that environmental factors can directly affect developmental rate, with temperature perhaps being the best studied and understood for the widest variety of taxa [33-36].

We previously demonstrated that within a cohort of synchronously fertilised *H. asinina *individuals some larvae achieve competence at an earlier age than others despite uniform environmental conditions [4]. Here we demonstrate that variation in developmental rate is manifested during early cell cleavages, with the discrepancy between slow and fast developing larvae increasing as development proceeds.

## Results

### Variation in the time to onset of 3rd cleavage

**Figure 1:**
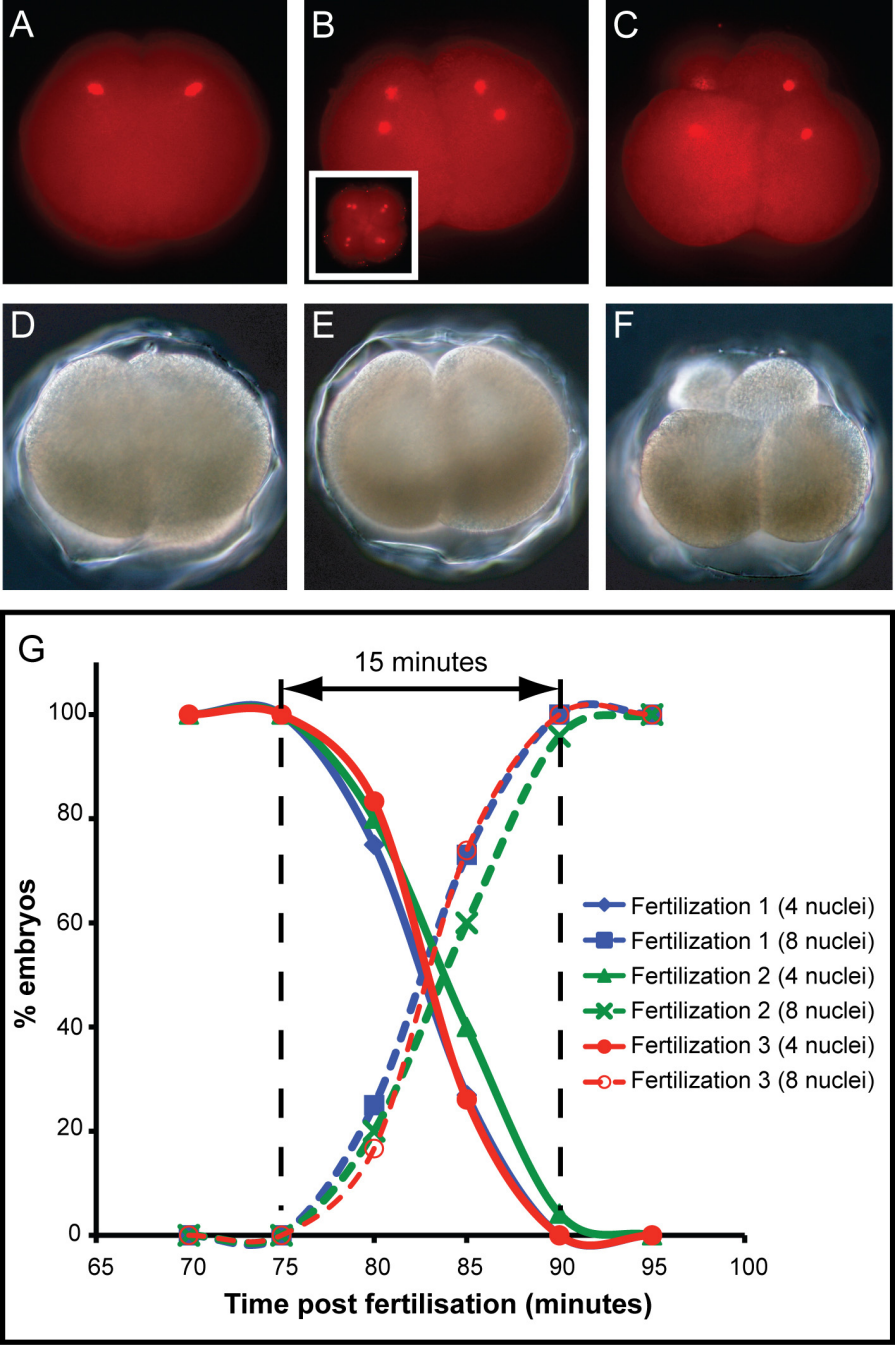
**Variation in the rate of third cleavage revealed by nuclear staining**. (A - F) Propidium iodide staining of early *H. asinina *embryos allowed the number of discrete nuclei to be accurately determined. All views are lateral except the inset in (B) which is from the animal pole. (A) A four-cell embryo with two distinct nuclei in the focal plane (four nuclei in total). (B) A morphologically similar embryo to that shown in (A) with four nuclei in the focal plane. When the same embryo is viewed from the animal pole (inset) eight nuclei are visible. Embryos shown in (A) and (B) are of the same chronological age. (C) Following cytokinesis, eight distinct cells can be seen with four of the eight nuclei visible in this focal plane. (D-F) Bright field views of the corresponding embryos as in (A-C). (G) Embryos from three unique fertilizations were monitored at five-minute intervals for the time taken to progress from four discrete nuclei to eight discrete nuclei. Each point represents a minimum of 20 observations

Variation in the rate at which embryos progressed from 4 to 8 cells (as defined by the number of distinct nuclei) was observed with the nuclear stain propidium iodide (Figure 1A-F). Using intervals of 5 min between observations, we found that a period of 15 min was required for all sampled embryos to complete the third round of anaphase, spanning from 75 to 90 min post-fertilization (Figure 1G). This pattern was observed across three independent fertilizations.

### Variation in the time to hatching

**Figure 2:**
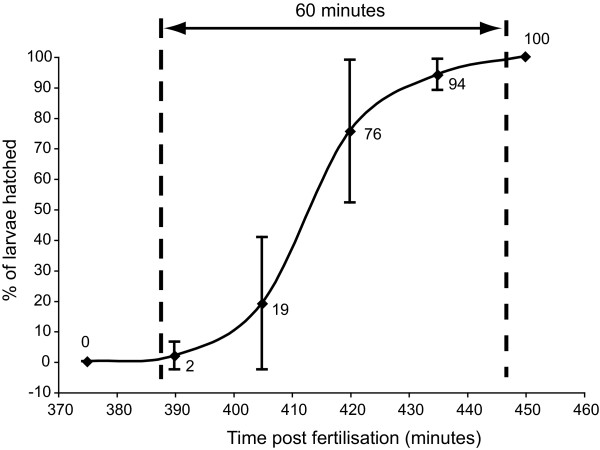
**Variation in the age at hatching from the vitelline envelope**. The age at which larvae derived from a single and synchronous fertilisation event hatched from the vitelline envelope was monitored in 15 minute intervals. Six replicates of 20 larvae from this fertilisation were monitored. Error bars are one standard deviation of the mean

Observing the time taken for every individual larva (from a cohort of synchronously fertilised eggs) to hatch from the vitelline envelope revealed a larger degree of chronological variation. A small proportion of individuals (2%) hatched from the vitelline envelope by 5.5 h post-fertilization (hpf), while it took 7.0 hpf for over 75% of individuals to hatch, and 7.5 hpf for every individual to emerge from the vitelline envelope (Figure 2).

### Variation in the rate of larval shell development revealed by Has-Ubfm1 expression

**Figure 3:**
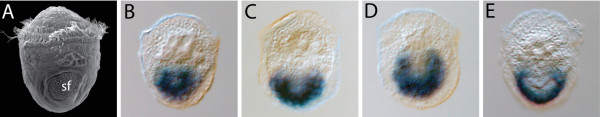
**Variation in rates of larval shell deposition between larvae of the same age (10 hpf) as revealed by *in situ *hybridisation against *Has-Ubfm *[GenBank:**DW986191**]**. (A) An SEM image of a *H. asinina *trochophore reveals the position of the shell field (sf). (B - D) Representative variation in the spatial expression of *Has-Ubfm*

The expression of the gene *Has-Ubfm1 *[GenBank:DW986191] is detected in the lateral and posterior periphery of the evaginated shell field of young trochophore larvae (Figure 3 and [37]). Normal shell development is initiated as a thickening and then an invagination of the dorsal ectoderm of the trochophore larva. Organic material (likely the primordial periostracum) is secreted by these cells. This dorsal ectoderm then evaginates to form the shell field which then expands in all directions concomitantly with calcification of the periostracum and formation of the larval shell field [38]. Among a sample of *H. asinina *larvae of exactly the same chronological age (10 hpf), a range of stages of shell development can be observed (Figure 3B-E). In some individuals, *Has-Ubfm1 *expression is localized to the periphery of the recently evaginated shell gland (Figure 3B), and thus appears as an almost a uniform field of expression. In other individuals of the same age, *Has-Ubfm1 *expression already has expanded such that a well-defined aperture, completely devoid of cells expressing *Has-Ubfm1*, can be identified (Figure 3E). While variation in the intensity of staining patterns generated by *in situ *hybridisation experiments are a well known phenomena for model organisms, here we are primarily interested in the spatial variation in gene expression rather than quantitative variation.

### Variation in late larval development - variable rates of metamorphosis amongst larvae of the same age

**Figure 4:**
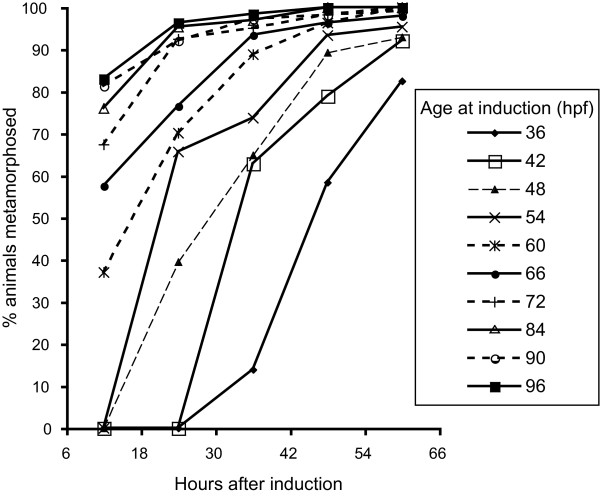
**Variation in the percentage of larvae of the same age initiating metamorphosis following induction by the crustose coralline alga (CCA) *Mastophora pacifica***. Larvae of various ages (36 - 96 hpf) were induced to metamorphose and their response, as indicated by postlarval shell growth, monitored at 12 h intervals from 12 to 60 hours after induction. Error bars are omitted for clarity of presentation. Note that the response to induction across all ages is never uniform. i.e. some larvae are able to respond to the CCA inductive cue faster than others

Significant variation in the percentage of larvae of the same age initiating metamorphosis (as observed by the initiation of post-larval shell growth) was observed after exposure to the inductive crustose coralline algae (CCA) *Mastophora pacifica *[4,39]. We commonly employ this method of scoring metamorphosis because more immediate responses to settlement inducing cues (such as velar abscission) are highly unreliable [4]. Because unambiguous post-larval shell growth takes at minimum several hours post-induction to observe, we report our results as the proportion of animals metamorphosed post-induction (Table 1 and Figure 4). The total larval age can be calculated by adding the time post induction to the age at induction. We have conducted experiments like these across multiple spawning events and multiple spawning seasons and consistently see the same pattern - some larvae are able to commence metamorphosis significantly earlier than others [4]. In this experiment, 37% of larvae aged 60 hpf at the time of induction (72 h total age) had initiated metamorphosis 12 h after induction. In contrast, 83% of larvae aged 96 hpf at the time of induction (108 h total age) showed signs of having initiated metamorphosis 12 h after induction (Table 1 and Figure 4).

**Table 1:** Proportion (% ± SD) of metamorphosed *H.asinina *following induction by the crustose coralline algae *Mastophora pacifica *as a function of age

	Hours after induction				
Age at induction (hours)	12	24	36	48	60
**36**	0.0 ± 0	0.0 ± 0	13.9 ± 22	58.4 ± 15	82.5 ± 20
**42**	0.0 ± 0	0.0 ± 0	62.9 ± 20	78.9 ± 15	92.1 ± 5
**48**	0.0 ± 0	39.5 ± 24	64.9 ± 8	89.3 ± 4	92.9 ± 5
**54**	0.0 ± 0	65.7 ± 21	73.8 ± 10	93.4 ± 8	95.4 ± 5
**60**	37.1 ± 23	70.2 ± 13	88.8 ± 5	96.4 ± 4	100.0 ± 0
**66**	57.6 ± 18	76.6 ± 12	93.5 ± 6	96.4 ± 3	98.0 ± 3
**72**	67.4 ± 21	92.5 ± 6	95.1 ± 5	98.3 ± 3	100.0 ± 0
**84**	76.1 ± 15	95.4 ± 5	97.0 ± 3	100.0 ± 0	100.0 ± 0
**90**	81.3 ± 6	92.1 ± 4	97.3 ± 3	98.2 ± 3	99.2 ± 2
**96**	83.0 ± 9	96.4 ± 3	98.4 ± 3	100.0 ± 0	100.0 ± 0

## Discussion

In *H. asinina*, as in other marine invertebrates, intra-cohort variation in rates of settlement and metamorphosis are commonly observed [5,23]. We have previously shown that larval age is not a good predictor of whether an individual will initiate metamorphosis when presented with an appropriate cue [4]. Here we show that variation in rates of embryogenesis and larval development may explain a proportion of the variation in the age at which competence is acquired, and hence the typically variable rates of metamorphosis observed within and between larval cohorts of the same age. Variation in the chronology of early embryological and larval developmental events (early cell divisions, hatching, rate of larval shell deposition) suggests that from fertilisation, idiosyncratic rates of development are present within a single cohort of synchronously fertilised, and genetically related (full sibling) *H. asinina *larvae. Differences in the rate of development produce larvae that display broader and broader ranges of developmental states as ontogeny proceeds; individuals complete early developmental events (3^rd ^cleavage and hatching) within narrower time spans (15 min and 60 min respectively) than the time taken for all individuals to undergo metamorphosis (36+ h). Because we have used a variety of non-equivalent characters (cleavage, hatching, gene expression and competency) at different time points we have not attempted to quantify this increasing discrepancy through development. To do this in a meaningful way, a character (ideally several) that can be accurately quantified on individual larvae at all stages of development first needs to be identified. Additionally, with the methods we have employed we cannot exclude the possibility that larvae which initially develop rapidly, may slow their rate of development and be among the last to acquire competence. Nonetheless the final outcome is the same; the total discrepancy between chronological age vs. developmental stage broadens as development proceeds. These results should also be considered in light of the fact that early developmental events are completed in less time than later events; a single cell division (third cleavage) takes on the order of minutes to complete, while the acquisition of competence requires hours to develop. The endpoint to this discrepancy is hinted at in the time taken for all larvae in a synchronously fertilised cohort to complete metamorphosis; given enough time, all larvae will eventually become competent to settle and metamorphose, as suggested by the asymptote-like characteristic of the curves in Figure 4. Conceptually extending these results to the field (albeit in the presence of unrealistic uniform oceanographic processes) would see larvae that take more time to attain competence settle and metamorphose a greater distance from the parent reef than larvae that develop more rapidly.

Although we provide evidence for variation in rates of development among full siblings of *H. asinina*, we cannot account for the mechanism that generates it. One potential epigenetic source could be variation in maternal provisioning. Indeed, such maternal effects have been shown to significantly influence larval and post-larval performance. For example Marshall and Keough demonstrated that larval size, a rough proxy for maternal provisioning in lecithotrophic larvae, can influence survival to adulthood and post-metamorphic growth [11]. However these affects diminish following metamorphosis and depend upon the environmental context the experiment is conducted in. Furthermore, that study examined larvae of mixed parentage, and investigated a species whose larval size (volume) varies by as much as 2.5 fold [11]. By comparing full siblings (see materials and methods) we reduce the impact of such maternal effects. Furthermore, eggs spawned by *H. asinina *vary in their diameter by less than 1.2 fold (1.2, 1.1 and 1.1 fold for three independent cohorts). We also demonstrate that much of the calculated egg volume variation is the result of systematic error (Table 2). If we assume that variation in maternal provisioning across a cohort of *H. asinina *gametes is minimal (at least significantly less than in those species for which maternal provisioning is known to affect larval and juvenile performance), then what is the source of the variation we report here? While our data cannot identify the source of this variation, it is tempting to speculate that a proportion of it may be genetically encoded, as recently suggested for a gastropod by Tills et al. [40]. Indeed variation in growth is known to be a heritable trait for juvenile *H. asinina *[41], a phenomenon that poses a significant challenge for efficient aquaculture practises. We propose that this post-metamorphic variation is simply an extension of the pre-metamorphic variation in developmental rate that we report here. Importantly, any mechanism that produces a cohort of larvae that develops at varying rates may have significant ecological consequences as it could provide a means of ensuring that progeny will attain competency at variable times and distances from the location of conception. The developmental state in which a given larva encounters a suitable inductive cue can have a profound impact on the process of metamorphosis and post-metamorphic performance. For abalone if it is encountered too early, a habituation-like response is generated [4,42], and may result in subsequent encounters with a suitable cue being ignored. If it is encountered too late, post-metamorphic growth and survival is compromised [43]. In the case of *H. asinina *it could therefore be advantageous to generate a cohort of larvae that encounter suitable settlement environments in a range of developmental states at varying distances from the point of inception. The observation that larvae from various phyla display variable rates of metamorphosis suggests that this strategy may be commonly employed [23,44,45]. If the source of this variation is in part genetically encoded, the identification of the molecular mechanisms that generate and/or maintain this variation would be an exciting contribution to our understanding of the way in which marine invertebrates are able to colonise patchily distributed habitats. Identification of the genes that encode hatching enzymes, receptors and/or signal transducers for metamorphosis inducing cues and other stage specific molecules would be a first step towards this goal.

**Table 2:** Estimates of inter- and intra-cohort egg volume variation in *H.asinina *and systematic error

	n	Mean diameter (μm)	**Std. dev**. (μm)	Min/Max (μm)	**Mean vol**. (μm^3^)	**Std. Dev**. (μm^3^)	Std. Error ^1^ (μm^3^)	Systematic error (%) ^2^
**Cohort 1 measurement 1**	46	136.0	4.2	120.0/144.4	1,321,897.9	117,418.7		35.6
**Cohort 1 measurement 2**	46	133.5	4.5	114.0/144.4	1,249,694.1	121,253.2		34.5
**Cohort 1 measurement 3**	46	133.5	4.2	117.2/141.1	1,249,108.2	112,218.9	41,857.1	37.3
**Cohort 2 measurement 1**	25	140.9	3.6	132.0/146.2	1,466,635.5	112,840.8		31.4
**Cohort 2 measurement 2**	25	138.9	2.9	132.0/144.8	1,404,027.4	88,846.1		39.9
**Cohort 2 measurement 3**	25	138.9	4.2	129.2/148.5	1,406,434.3	126,670.2	35,472.4	28.0
**Cohort 3 measurement 1**	27	138.0	4.7	129.7/147.6	1,381,673.6	142,105.0		15.8
**Cohort 3 measurement 2**	27	136.6	3.6	131.0/143.9	1,336,833.8	105,865.6		21.2
**Cohort 3 measurement 3**	27	137.3	4.7	126.4/146.2	1,360,755.8	136,909.9	22,436.7	16.4

**^1 ^**The standard error is the standard deviation of sample means reported in column 6 (Mean vol.)

^2 ^The systematic error is the fraction of the standard deviation accounted for by the standard error (i.e. the variation obtained by measuring the same eggs 3 times as a proportion of the estimated variation in egg volume). This was calculated as the Std. Error (column 8) divided by the Std. Dev. (column 7) expressed as a percentage. Note that these values can only be underestimates of the total systematic error, additional sources of error will inflate these values

More than 35 years ago Strathmann reviewed the phenomenon of variation (spread) among cohorts of sibling marine invertebrate larvae, and concluded that "...there is probably a bias in the literature against records of variability in behaviour or rates of development. Most marine biologists have been unaware of the possible adaptive significance of spreading siblings, so the investigator is likely to note only the mode, mean, minimum, or maximum, depending on which is felt to represent natural conditions." [5]. Since this time mechanisms of larval dispersal and recruitment have received increased attention because these processes are known to directly affect adult population structures (for example see [46,47] however see [48,49] for exceptions). Variation in the timing of competence acquisition could serve to increase the spatial selection by larvae of distinct settlement sites, thereby minimising the chances of extinction due to local catastrophes [50-52]. While the data we present here may play a role in dispersal (and other) processes, we cannot say to what degree they do so. Many other factors are well known to affect the dispersal patterns of broadcast spawned gametes, for example currents and tidal movements [53], maternal provisioning [54] and paternal affects [55]. To quantify the contribution that variable rates of development might make to the fitness of planktonically dispersing organisms remains an exciting challenge for future marine ecologists, population geneticists and developmental biologists.

## Conclusions

Using a variety of methods we have demonstrated that substantial differences in the rate of developmental can be observed between individual *H. asinina *larvae originating from a synchronous, full sibling fertilisation event. The discrepancy between developmental state and chronological age appears to increase as development proceeds, and is likely to be a factor that contributes to the variation in rates of metamorphosis commonly observed within and between similar aged cohorts of abalone larvae.

## Methods

### Production of embryos and larvae

All gametes were produced from natural spawnings of *H. asinina *following methods previously described [4,56]. Gravid broodstock were maintained in individual spawning aquaria in order to control for the effects of parentage (and therefore differing nutritional histories between individuals) and timing of each fertilisation event. Six mature individuals (3 females and 3 males) were used to perform three unique and independent fertilization events. Sperm was added to unfertilized eggs, gently but thoroughly mixed, allowed to stand for 1 minute, and was then thoroughly washed out to reduce the chances of delayed fertilization events. By minimising the insemination time in this way, non-synchronous fertilisation events (± 1 min) were eliminated. For each experiment described, a sample of unfertilized eggs were set aside from each spawning event to monitor for signs of cleavage as an indicator of unsolicited fertilization. Zygotes from each fertilization were maintained in separate aquaria as previously described [4,41].

### Measuring variation in the rate of 3rd cleavage

Embryos from each of the three fertilization events were allowed to develop for 60 min after which approximately 100 individuals were placed in 4% paraformaldehyde in 0.2 μm filtered seawater (FSW) for approximately 10 min. Embryos were then briefly washed with FSW 3 times, phosphate buffered saline (PBS) 3 times, and then stored in PBS with 0.1% Tween (PBTw) at 4°C until further processing. This sampling procedure was repeated every 5 min from 60 to 100 min post fertilization (mpf). Nuclei were subsequently stained with 10 μg/ml propidium iodide in PBS for 0.5 - 2 h at room temperature, and were washed with PBTw 3 times over 30 min. Embryos were mounted in 60% glycerol in PBS and viewed under standard fluorescent optics. For each time point a minimum of 20 embryos from each fertilization were viewed laterally and from the animal pole and scored for the number of discrete nuclei.

### Variation in the time taken to hatch

Six replicates of 20 synchronously fertilized, normally cleaving embryos derived from a single cross, were reared in 10 ml FSW in a 6 well tissue culture dish at approximately 28°C for 6 h. Observations were then made every 15 minutes for hatched, swimming larvae. At each time point, all hatched and swimming larvae were counted and removed, with care taken to avoid physical disturbance of unhatched larvae.

### Variation in spatial gene expression during development

A genetic marker was used to monitor the variation in development of *H. asinina *trochophores. Following an EST screen for genes involved in shell formation [37,57], we uncovered a gene (*Has-Ubfm1*, GenBank accession number DW986191) that is expressed in the developing shell field of the trochophore larva. Using an *in situ *hybridisation probe against *Has-Ubfm1 *we found we could make relative comparisons of larval shell development.

For each whole mount *in situ *hybridization (WMISH) experiment thousands of actively swimming, morphologically normal larvae from a synchronous fertilization event derived from a single cross were collected and fixed and processed as previously described [57]. Although the aim of these experiments was not to quantitate gene expression, the colour reaction was terminated synchronously to allow direct comparisons between individual larvae to be made. Larvae were dehydrated through an ethanol series, mounted and cleared in benzyl benzoate:benzyl alcohol, and viewed under Nomarski optics with an Olympus DP10 digital camera connected to a BX40 microscope.

### Settlement assays

Settlement assays were performed as described in [4]. Briefly, embryos derived from a single cross were allowed to develop in 10 litres of static 5 μm filtered seawater. Hatched and normal swimming larvae were collected approximately 9 h later and transferred into a 300 mm diameter culture chamber with a 190 μm screen floor and flowing 5 μm filtered seawater. Larvae were maintained in this way until used in settlement assays. Beginning at 36 hpf and at 6 h intervals thereafter up to 96 hpf, a sample of 120 larvae was divided into 6 replicates (20 larvae/replicate) and were exposed to the CCA identified as *Mastophora pacifica *[4,39]. Careful attention was paid to ensure that every larva contacted the CCA when introduced into the experiment. Observations of metamorphosis were made 12 h after induction at 12 h intervals to 60 h after induction. Larvae were visually inspected under a dissecting microscope for signs of postlarval shell growth, an unambiguous morphological marker of metamorphosis [4].

### Egg volume measurements

Three cohorts of unfertilised eggs from 3 separate mothers were collected and photographs of individual eggs taken using an Olympus DP10 digital camera connected to a BX40 microscope. Individual egg diameters were measured independently 3 times using Macnification v1.7.1 [58]. Means, standard deviations and standard errors were calculated using Excel for Mac 2008 v 12.2.4.

## Competing interests

The authors declare that they have no competing interests.

## Authors' contributions

DJJ conducted the experimental work and drafted the manuscript. DJJ, SMD and BMD conceived the study and revised the manuscript. All authors read and approved the final manuscript.
